# Effects of blood pressure and antihypertensive drugs on osteoarthritis: a mendelian randomized study

**DOI:** 10.1007/s40520-023-02530-8

**Published:** 2023-08-21

**Authors:** Yinzhen Zhang, Yanpeng Wang, Changwei Zhao, Wenjun Cai, Zhengyan Wang, Wenhai Zhao

**Affiliations:** 1 Department of Traditional Chinese Medicine, Changchun University of Chinese Medicine, Changchun, China; 2 Department of Orthopedics, Affiliated Hospital of Changchun University of Chinese Medicine, Changchun, China; 3 Department of Orthopedics, The Third Affiliated Clinical Hospital of Changchun University of Chinese Medicine, Changchun, China

**Keywords:** PSDs and aldosterone antagonist, Osteoarthritis, Hypertension, Mendelian randomization, Drug targets

## Abstract

**Background:**

Previous studies have suggested that antihypertensive drugs may play a role in the treatment of osteoarthritis, but these studies may be limited by confounding factors and lead to biased results. Therefore, we conducted a Mendelian randomization study to investigate the effects of blood pressure and antihypertensive drugs on osteoarthritis.

**Methods:**

We used published large-scale genome-wide association data and applied univariate and multivariate Mendelian randomization methods. The main analysis model was inverse variance weighting, and the reliability of the results was tested using MR-Egger intercept analysis, Cochran's *Q* test, and leave-one-out analysis. We comprehensively evaluated the relationship between systolic blood pressure, diastolic blood pressure, 12 antihypertensive drugs, and osteoarthritis. We also conducted verification in the independent queue of UK Biobank and built a simple linear regression model to obtain an independent comparison.

**Results:**

We found no evidence that systolic and diastolic blood pressure significantly affected osteoarthritis. However, among antihypertensive drugs, we observed a significant positive correlation between potassium-preserving diuretics and aldosterone antagonists and all osteoarthritis (OR: 0.560, 95% CI 0.406–0.772, *P* = 0.0004). Sensitivity analysis showed no horizontal pleiotropy or heterogeneity, and the leave-one-out analysis demonstrated the reliability of the results. This result was replicated with nominally statistical significance in the validation cohort and exhibited significant correlation in the linear regression analysis.

**Conclusions:**

Our study suggested that controlling the protein targets of potassium-sparing diuretics and aldosterone antagonists may have beneficial results for osteoarthritis. These findings provide valuable medication strategies for the control of hypertension in patients with osteoarthritis.

**Supplementary Information:**

The online version contains supplementary material available at 10.1007/s40520-023-02530-8.

## Introduction

Osteoarthritis(OA) is a degenerative joint disease related to cartilage that affects approximately 300 million people worldwide [1]. It predominantly affects the knee, hand, and hip [2], resulting in chronic pain, joint stiffness, and mobility disorders that significantly affect patient’s quality of life. Various risk factors contribute to OA, including age, smoking, body mass index(BMI), low-density lipoprotein(LDL), and alcohol consumption, previous studies have also revealed the role of hypertension in the development of knee OA [3–5]. Therefore, controlling blood pressure may be a crucial factor in preventing OA.

Previous studies suggest that the homeostasis regulation of chondrocytes depends on the local renin–angiotensin system. Thus, angiotensin-converting enzyme (ACE) inhibitors or angiotensin-II receptor blockers may have therapeutic potential for knee OA [6]. Li et al. found that the use of calcium channel blockers is associated with a narrowed knee joint space [7], while Driban et al. observed that thiazides are correlated with changes in symptoms in patients with knee OA [8]. Although various studies have indicated the value of antihypertensive drugs in the prevention and treatment of OA, sample size and experimental design limitations have made it challenging to establish causal relationships between drug use and changes in OA-related traits. Moreover, observational studies are susceptible to mixed factors and reverse causal relationships, leading to biased results. Mendelian randomization(MR) provides a novel approach to address these issues.

Mendelian randomization is a statistical method that uses genetic variation as an instrumental variable to estimate the causal relationship between exposure and outcome [9]. Because genetic variation is randomly distributed during conception, it is unlikely to be influenced by environmental and confounding factors, thereby minimizing confounding bias and providing robust evidence of causal relationships. Drug-targeted Mendelian randomization is a research method that uses drug target-related genetic variation to simulate drug effects. Several studies have successfully evaluated the relationship between antihypertensive drugs and Alzheimer's disease [10, 11] and other diseases [12, 13]. In this study, we used data from published studies as instrumental variables to comprehensively evaluate the relationship between antihypertensive drugs and OA and assess their potential therapeutic value.

## Methods and design

### Research design

We conducted a two-sample Mendelian randomized method. Initially, we assessed the causal relationship between systolic blood pressure(SBP) and diastolic blood pressure(DBP) and all OA, hand OA, hip OA, and knee OA. Next, we employed the multivariate Mendelian randomized(MVMR) study method to examine the impact of blood pressure on these four types of OA after multivariate adjustment. Finally, we utilized published genetic variation of antihypertensive drug targets as instrumental variables to evaluate the effects of 12 types of antihypertensive drugs on OA. These drugs included alpha-adrenoceptor blockers, angiotensin-converting enzyme inhibitors, angiotensin-II receptor blockers, beta-adrenoceptor blockers, calcium channel blockers (CCB), centrally acting antihypertensive drugs, loop diuretics, potassium-sparing diuretics and aldosterone antagonists, renin inhibitors, thiazides and related diuretics, and vasodilator antihypertensives. We also used the independent cohort of osteoarthritis in the UK Biobank to conduct a validation analysis.

### Selection of data sources and instrumental variables

#### Systolic and diastolic blood pressure

We obtained systolic and diastolic blood pressure phenotypes from the IEU Open GWAS (DBP: ukb-a-359, SBP: ukb-a-360), which included 317,756 samples from European populations. We selected single nucleotide polymorphisms(SNPs) closely related to DBP and SBP (*p* < 5 × 10^–8^) and removed those in linkage disequilibrium (*r*^2^ = 0.001, genetic distance = 10 MB). We then eliminated SNPs related to OA, BMI, smoking, obesity, drinking, and low density lipoprotein (LDL) through the Phenoscanner data website (http://www.phenoscanner.medschl.cam.ac.uk/) [14]. We calculated the F value of each remaining SNP and removed those with an F value less than 10. Finally, we used the MR-PRESSO method to screen and eliminate outliers and used the remaining SNPs as the final instrumental variables.

#### Antihypertensive drugs

We selected 12 genetic proxies for antihypertensive drugs from previously published studies [15]. In this research, authors initially extracted protein targets and genes of 12 antihypertensive drugs from the Drugbank database, following which they screened the corresponding gene's "best SNP" in each tissue using the GTeX project data. After conducting two-sample MR and performing column quality control, the researchers selected SNPs that significantly affected SBP as the final analysis SNP. The detailed process of SNP screening is available in the original study by the authors [15]. To circumvent confounding factors, we scrutinized and eliminated SNPs linked to OA, BMI, obesity, smoking, drinking, and LDL in the Phenoscanner. We further employed the MR-PRESSO method to screen and discard any outliers.

#### Outcome

We extracted OA GWAS data from the Genetics of Osteoarthritis (GO) Consortium [16], which included 177,517 OA cases. Hand OA, hip OA, and knee OA were 20,901 cases, 36,445 cases, and 62,497 cases, respectively, all of which were European population samples. The GWAS data of the validation cohort is from the UK Biobank. Due to the lack of GWAS data of hand Osteoarthritis, we only conducted validation analysis on all OA, knee OA and hip OA in the outcome.

### Statistical analysis

We utilized three different statistical methods: inverse variance weighting(IVW), weighted median estimator (WME), and MR Egger regression. Specifically, IVW was the primary analysis method, while the other two methods supplemented our analysis. Heterogeneity was detected using Cochran's Q test. We evaluated the bias of gene pleiotropy using MR Egger intercept analysis. If the regression intercept is closer to 0, gene pleiotropy is less likely to have occurred. We also used a sensitivity analysis known as leave-one-out. We replicated the above method for the verification cohort. To ensure independent comparison based on MR, we established a machine learning model using the harmonized data. For the analysis, we employed the linear regression model in this test set. To examine the impact of effect size on the outcome of exposure factors, we randomly split the data into a training set (80%) and a test set (20%). We considered a *p* value less than 0.05 as statistically significant.

All analyses were conducted using RStudio 2023.03.1, R 4.22, TwoSample MR, and the MR PRESSO package. In univariate MR analysis of blood pressure and OA, we considered a *p* value of < 0.006 (0.05/8) to be significantly different after Bonferroni correction. In MVMR, we considered a *p* value of < 0.05 to be significantly different. In antihypertensive drugs and OA analysis, a *p* value of < 0.001(0.05/48) was considered to be significantly different. Any *p* value between 0.05 and significant results was considered to be nominally significant.

## Results

### Instrumental variables

Following screening, we employed 168, 157, and 289 SNPs, respectively, as instrumental variables for DBP, SBP, and antihypertensive drugs. Further information regarding the instrumental variables can be accessed in the supplemental file.

### MR analysis of blood pressure and OA

**Fig. 1:**
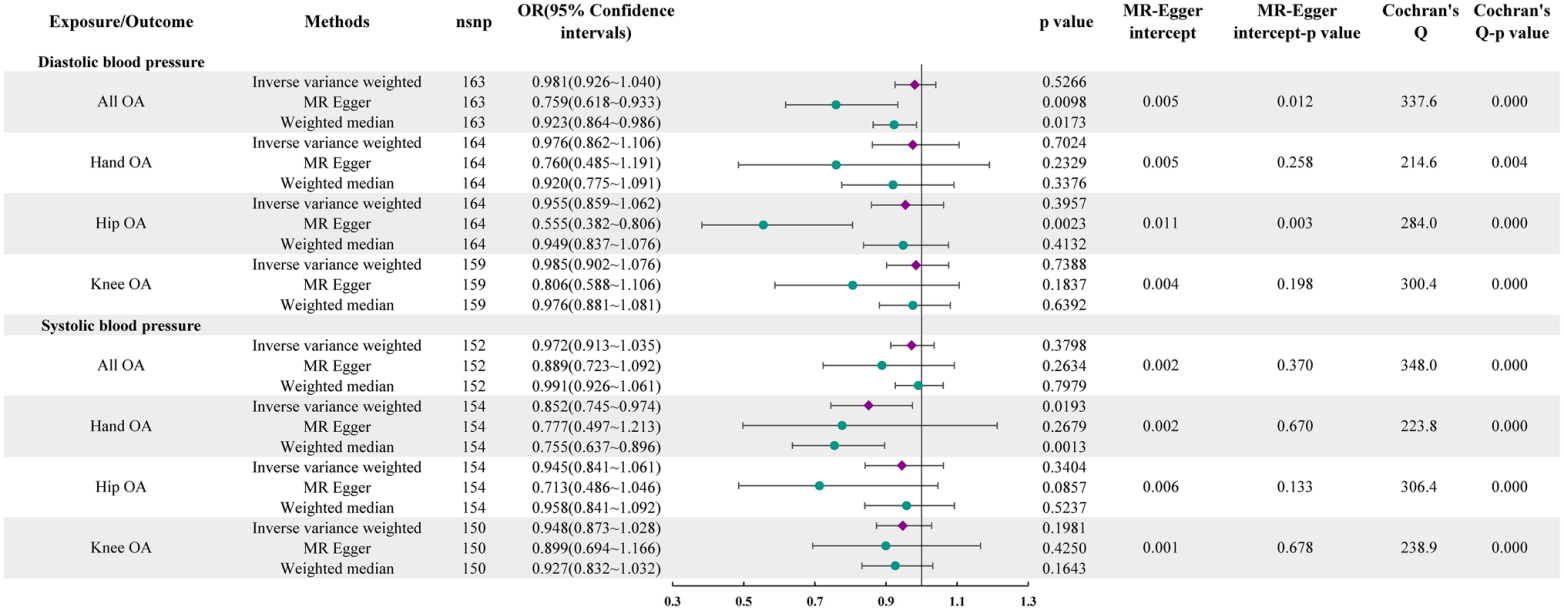
Univariate MR analysis results of blood pressure and OA

**Fig. 2:**
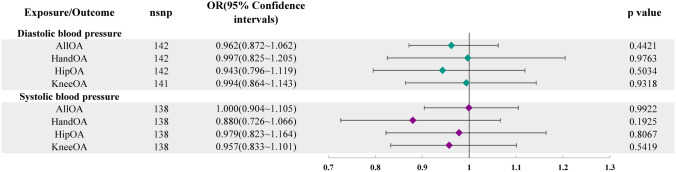
MR analysis results of the relationship between blood pressure and OA after multivariable adjustment

Figure 1 displays the results of the univariate MR analysis. Our primary IVW analysis did not identify evidence that blood pressure substantially influences OA. However, we observed nominally significant evidence of a relationship between SBP and hand OA, with an OR of 0.852 (95% CI 0.745–0.974, *P* = 0.0193). In the MVMR analysis in Fig. 2, we did not observe evidence that blood pressure influences OA.

### Antihypertensive drugs and OA

**Fig. 3:**
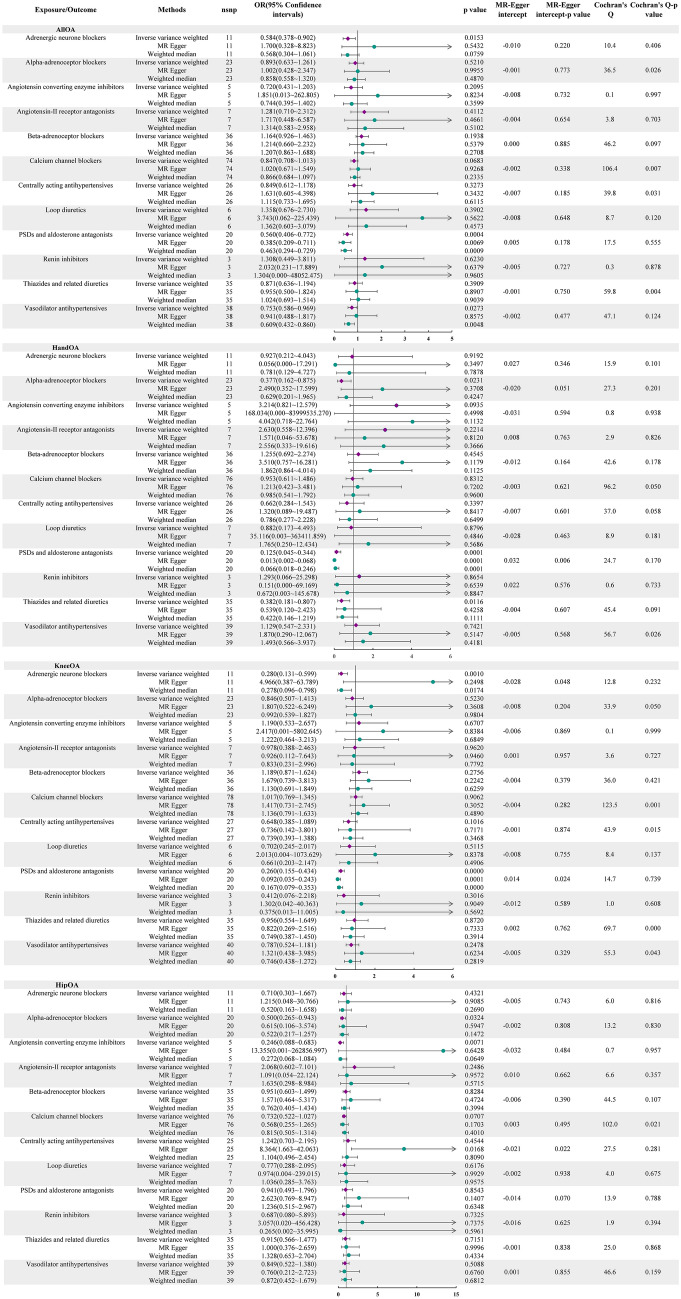
MR analysis results of the relationship between antihypertensive drugs and OA

In our analysis of the relationship between antihypertensive drugs and OA, we found significant evidence supporting a causal association between PSDs and aldosterone antagonists with all OA (OR: 0.560, 95% CI 0.406–0.772, *P* = 0.0004), as well as hand OA (OR: 0.125, 95% CI 0.045–0.344, *P* = 0.0001) and knee OA (OR: 0.260, 95% CI 0.155–0.434, *P* = 0.0000) in our primary analysis. Consistent and significant or suggestive results were also observed in the other two methods. Specifically, we also observed a significant relationship between adrenergic neurone blockers and knee OA in our primary analysis (OR: 0.280, 95% CI 0.131–0.599, *P* = 0.001) (Fig. 3).

**Fig. 4:**
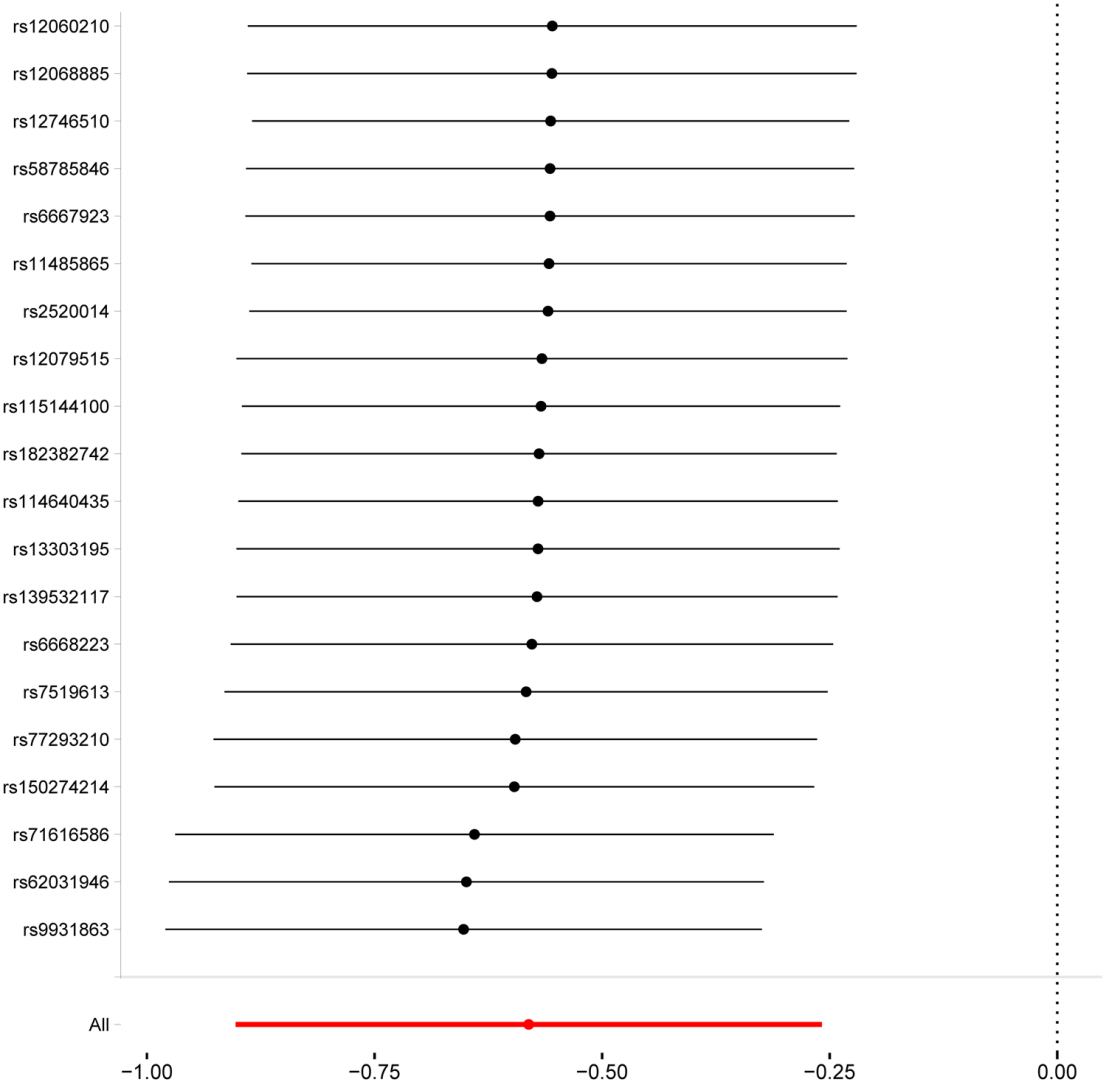
MR leave−one−out sensitivity analysis for PSDs and aldosterone antagonists on AllOA

In our sensitivity analysis, we observed horizontal pleiotropic effects during the analysis of PSD and aldosterone antagonists for both hand and knee OA, as well as during the analysis of Adrenergic neurone blockers for knee OA. However, we found no such effects or heterogeneity during the MR analysis of PSDs and aldosterone antagonists with all OA (Fig. 3). In addition, our leave-one-out analysis further supports the reliability of our results (Fig. 4).

### Cohort validation

No evidence of a causal relationship between blood pressure and osteoarthritis was observed in the validation cohort. However, extensive heterogeneity was found in the sensitivity analysis (Table 5 of the supplementary). Among antihypertensive drugs, we identified a significant causal relationship between calcium channel blockers and all OA (OR: 1.038, 95% CI 1.019–1.059, *P* = 0.0001). We also found a significant causal relationship between PSDs and knee OA (OR: 0.184, 95% CI 0.089–0.378, *P* = 0.0000). However, horizontal pleiotropy was observed in the MR analysis of PSDs and aldosterone antagonists with knee OA (*P* = 0.013). Furthermore, a nominally significant causal relationship was found between PSDs and aldosterone antagonists with all OA (OR: 0.935, 95% CI 0.892–0.980, *P* = 0.0055) and hip OA (OR: 2.244, 95% CI 1.008–4.995, *P* = 0.0478). The MR analysis of PSDs and aldosterone antagonists with hip OA also revealed horizontal pleiotropy (*P* = 0.013). In addition, a nominally significant causal relationship was found between loop diuretics and knee OA (OR: 0.149, 95% CI 0.035–0.643, *P* = 0.0107). The results of the validation cohort are presented in Table 6 of the supplementary.

### Linear regression results

The linear regression results provide evidence for the protective effect of PSDs and aldosterone antagonists on all OA (*β* = −0.431, *P* = 0.000). Furthermore, the regression results also indicate a protective effect of PSDs and aldosterone antagonists on hand OA and knee OA. Moreover, our results demonstrated the influence of antihypertensive drugs, such as calcium channel blockers and beta adrenoceptor blockers, on OA. Detailed linear regression results are provided in Table 7 of supplementary.

## Discussion

Our MR study did not yield any evidence to suggest a significant association between blood pressure and OA. However, we identified a causal relationship between PSDs and aldosterone antagonists in relation to all OA, indicating a protective effect against OA. This result was nominally replicated with statistical significance in the validation data set and exhibited significant correlation in the linear regression analysis. Furthermore, our sensitivity analysis provides additional support for the reliability of these findings.

The relationship between blood pressure and OA has been extensively studied. A previous study found a gender difference in the association between hypertension and the imaging of OA [3]. In addition, a Mendelian randomization study showed that SBP was associated with all OA, hip OA and knee OA [17]. However, a cross-sectional study conducted in South Korea showed that hypertension was negatively correlated with the prevalence of OA [18], which may be related to the continuous use of antihypertensive drugs. Wang et al.'s research [19] revealed a correlation between blood pressure and arterial stiffness at various locations, as well as the volume of knee cartilage, suggesting that blood pressure may affect the progression of OA. Despite several studies indicating a relationship between blood pressure and OA, our analysis did not find significant evidence supporting the idea that blood pressure affects OA. However, specific antihypertensive drugs were found to have a protective effect on OA, which suggests that they may play a role in protecting against OA by means other than simply reducing blood pressure.

In our study, PSDs and aldosterone antagonists were thoroughly validated and found to have a causal relationship with OA. However, previous studies have also indicated a potential association between other antihypertensive drugs and OA. A study using data from the Osteoarthritis initiative demonstrated that CCB may exacerbate pain in patients with OA. The findings indicate that, during the 4-year follow-up period, the pain score of the CCB group was higher compared to other antihypertensive drugs. Although no statistical difference was observed, the CCB group exhibited the narrowest joint gap across multiple test groups [7]. In another study [20], it was found that Nifedipine, a classic CCB, may have a detrimental effect on chondrocyte proliferation. In addition, previous studies [7, 21, 22] have suggested that beta-adrenoceptor blockers can alleviate joint pain. Furthermore, a systemic review [23] on losartan and Angiotensin-II receptor blockers indicates that Angiotensin-II receptor blockers have beneficial effects on chondrocytes in animals, suggesting a potential protective effect against Osteoarthritis.

OA is considered to be closely associated with a series of inflammatory reactions. Prior research has demonstrated that Aldosterone plays a role in promoting inflammatory effects [24]. Consequently, blocking Aldosterone may be beneficial in ameliorating the pain and other symptoms experienced by OA patients. Elsaman et al.’s investigation indicated that knee joint effusion related to OA can be improved using low-dose Spironolactone [25]. A previous randomized controlled trial (RCT) [26] also indicated significant improvement in quality of life among subjects who orally took 25 mg of Spironolactone every day. This improvement was demonstrated 5 month post-treatment, with about one-third of the Spironolactone group reporting pain and discomfort relief. Conversely, another proof-of-concept test involving 86 subjects based on this study found that taking Spironolactone does not significantly improve pain or quality of life among knee OA patients, whose average age surpassed 77 years old [27]. However, the study did indicate that taking 25 mg Spironolactone daily is highly safe for OA patients in this age group. Although our research demonstrates the potential efficacy of Aldosterone antagonists in treating OA, high-quality RCT verification is still necessary.

Our study's advantage is utilizing MR to investigate the effects of blood pressure and antihypertensive drugs on OA. By upholding MR's basic hypotheses, we minimized bias and confounding factors to a great extent. In addition, we employed publicly available data in conjunction with our research design to screen out instrumental variables, ensuring Instrumental Variables Estimation's maximum reliability. We further validated the results’ dependability via sensitivity analysis.

However, this research also has its limitations: first, the GWAS data we used was limited to individuals of European ancestry. Unfortunately, we could not explore the effects of blood pressure and antihypertensive drugs on other races, since sufficient data were not available. Second, the ability of Instrumental Variables Estimation to explain related genetic traits is limited. Finally, as MR studies primarily deal with assessing associated effects, additional high-quality research is needed to verify the improvement of antihypertensive drugs on OA.

## Conclusion

The genetic prediction of PSDs and aldosterone antagonists suggested their protective effects against OA, providing essential guidance for the redevelopment of current drugs and therapy for hypertension in osteoarthritic patients. Nonetheless, additional high-quality research is still necessary for confirmation.

### Supplementary Information

Below is the link to the electronic supplementary material.

Supplementary file (XLSX 114 KB)

## Data Availability

The data used in this article is included in the supplementary file. GWAS data of osteoarthritis can be found in Genetics of Osteoarthritis (GO) Consortium. (https://www.genetics-osteoarthritis.com), GWAS data of intermediary factors can be found in IEU Open GWAS. (https://gwas.mrcieu.ac.uk/).

## References

[1] Abramoff B, Caldera FE (2020). Osteoarthritis: pathology, diagnosis, and treatment options. Med Clin North Am.

[2] March LM, Bachmeier CJ (1997). Economics of osteoarthritis: a global perspective. Baillieres Clin Rheumatol.

[3] Lo K, Au M, Ni J (2022). Association between hypertension and osteoarthritis: a systematic review and meta-analysis of observational studies. J Orthop Translat.

[4] Shi X, Schlenk EA (2022). Association of hypertension with knee pain severity among people with knee osteoarthritis. Pain Manag Nurs.

[5] Zhang YM, Wang J, Liu XG (2017). Association between hypertension and risk of knee osteoarthritis: a meta-analysis of observational studies. Medicine (Baltimore).

[6] Zhou L, Kwoh CK, Ran D (2020). Lack of evidence that beta blocker use reduces knee pain, areas of joint pain, or analgesic use among individuals with symptomatic knee osteoarthritis. Osteoarthritis Cartilage.

[7] Li M, Zeng Y, Nie Y (2021). The effects of different antihypertensive drugs on pain and joint space width of knee osteoarthritis - A comparative study with data from Osteoarthritis Initiative. J Clin Hypertens (Greenwich).

[8] Driban JB, Lo GH, Eaton CB (2016). Exploratory analysis of osteoarthritis progression among medication users: data from the Osteoarthritis Initiative. Ther Adv Musculoskelet Dis.

[9] Huang X, Zhang T, Guo P (2023). Association of antihypertensive drugs with fracture and bone mineral density: a comprehensive drug-target Mendelian randomization study. Front Endocrinol (Lausanne).

[10] Chauquet S, Zhu Z, O'Donovan MC (2021). Association of antihypertensive drug target genes with psychiatric disorders: a mendelian randomization study. JAMA Psychiat.

[11] Ou YN, Yang YX, Shen XN (2021). Genetically determined blood pressure, antihypertensive medications, and risk of Alzheimer's disease: a Mendelian randomization study. Alzheimers Res Ther.

[12] Liu J, Li S, Hu Y (2022). Repurposing antihypertensive drugs for the prevention of glaucoma: a mendelian randomization study. Transl Vis Sci Technol.

[13] Zhao JV, Liu F, Schooling CM (2022). Using genetics to assess the association of commonly used antihypertensive drugs with diabetes, glycaemic traits and lipids: a trans-ancestry Mendelian randomisation study. Diabetologia.

[14] Kamat MA, Blackshaw JA, Young R (2019). PhenoScanner V2: an expanded tool for searching human genotype-phenotype associations. Bioinformatics.

[15] Walker VM, Kehoe PG, Martin RM (2020). Repurposing antihypertensive drugs for the prevention of Alzheimer's disease: a Mendelian randomization study. Int J Epidemiol.

[16] Boer CG, Hatzikotoulas K, Southam L (2021). Deciphering osteoarthritis genetics across 826,690 individuals from 9 populations. Cell.

[17] Funck-Brentano T, Nethander M, Moverare-Skrtic S (2019). Causal factors for knee, hip, and hand osteoarthritis: a mendelian randomization study in the UK biobank. Arthritis Rheumatol.

[18] Bae YH, Shin JS, Lee J (2015). Association between hypertension and the prevalence of low back pain and osteoarthritis in koreans: a cross-sectional study. PLoS One.

[19] Wang Y, Meng T, Ruan G (2021). Associations of blood pressure and arterial stiffness with knee cartilage volume in patients with knee osteoarthritis. Rheumatology (Oxford).

[20] Uzieliene I, Bernotiene E, Rakauskiene G (2019). The antihypertensive drug nifedipine modulates the metabolism of chondrocytes and human bone marrow-derived mesenchymal stem cells. Front Endocrinol (Lausanne).

[21] Martin LJ, Piltonen MH, Gauthier J (2015). Differences in the antinociceptive effects and binding properties of propranolol and bupranolol enantiomers. J Pain.

[22] Valdes AM, Abhishek A, Muir K (2017). Association of beta-blocker use with less prevalent joint pain and lower opioid requirement in people with osteoarthritis. Arthritis Care Res (Hoboken).

[23] Yamaura K, Nelson AL, Nishimura H (2023). The effects of losartan or angiotensin II receptor antagonists on cartilage: a systematic review. Osteoarthritis Cartilage.

[24] Bendtzen K, Hansen PR, Rieneck K, Spironolactone/Arthritis Study G (2003). Spironolactone inhibits production of proinflammatory cytokines, including tumour necrosis factor-alpha and interferon-gamma, and has potential in the treatment of arthritis. Clin Exp Immunol.

[25] Elsaman AM, Radwan AR, Mohammed WI (2016). Low-dose spironolactone: treatment for osteoarthritis-related knee effusion. A prospective clinical and sonographic-based study. J Rheumatol.

[26] Altman R, Asch E, Bloch D (1986). Development of criteria for the classification and reporting of osteoarthritis Classification of osteoarthritis of the knee Diagnostic and Therapeutic Criteria Committee of the American Rheumatism Association. Arthritis Rheum.

[27] McMurdo ME, Sumukadas D, Donnan PT (2016). Spironolactone for people age 70 years and older with osteoarthritic knee pain: a proof-of-concept trial. Arthritis Care Res (Hoboken).

